# Severe Iodine Deficiency–Associated Hypothyroidism in a Toddler With Cow’s Milk Allergy: A Case Report

**DOI:** 10.1155/crpe/8276700

**Published:** 2026-11-11

**Authors:** Judit Laidlaw, Lauren Rubin

**Affiliations:** ^1^ Department of Nutritional Sciences Cornell University Ithaca New York USA cornell.edu; ^2^ Department of Pediatrics Atrius Health Newton Massachusetts USA

## Abstract

Iodine is an essential micronutrient required for thyroid hormone synthesis and normal growth and neurodevelopment. Although iodine deficiency is uncommon in high‐income countries with iodization programs, children with restrictive diets may remain at risk. We report a 23‐month‐old male who presented with growth deceleration and was found to have severe hypothyroidism. Initial laboratory evaluation demonstrated low FT4 with markedly elevated thyroid‐stimulating hormone (> 200 mIU/L). Endocrine evaluation revealed diffuse thyroid enlargement with hypervascularity on ultrasonography and elevated thyroglobulin levels, with negative thyroid autoantibodies. Urinary iodine‐to‐creatinine ratio was low, consistent with iodine deficiency. Dietary review revealed multiple immunoglobulin E–mediated food allergies, including cow’s milk allergy, and reliance on commercially available allergy‐free foods lacking iodized salt. The patient was treated with levothyroxine, resulting in rapid normalization of thyroid function. Follow‐up demonstrated improvement in urinary iodine levels and resolution of thyroid enlargement. This case highlights iodine deficiency as a reversible cause of severe hypothyroidism and growth delay in young children with restrictive diets. Clinicians should consider iodine deficiency in toddlers with food allergies and growth abnormalities, even in regions where iodine sufficiency is presumed.

## 1. Introduction

Iodine is an essential micronutrient required for the synthesis of the thyroid hormones thyroxine (T4) and triiodothyronine (T3), which play critical roles in metabolism, growth, and neurodevelopment [1]. Thyroid hormone production is regulated by thyroid‐stimulating hormone (TSH), which stimulates the thyroid gland to synthesize and release T4 and T3. In the setting of iodine deficiency, impaired thyroid hormone synthesis leads to compensatory elevation of TSH and thyroid hypertrophy, often manifesting as goiter. Iodine deficiency has profound effects on growth and development and remains the leading cause of preventable intellectual disability worldwide [1].

To address global iodine deficiency, the World Health Organization recommended universal salt iodization in 1994. In the United States, the population iodine status improved following implementation of voluntary iodization programs, with data from the 2007 National Health and Nutrition Examination Survey indicating overall iodine sufficiency [2]. However, iodine intake remains highly dependent on dietary patterns, and personal food choices continue to influence the iodine status. The increased use of noniodized salts has contributed to reduced iodine intake in some populations.

In children and adolescents, approximately 53%–70% of dietary iodine intake is derived from dairy products, due to the presence of iodine in cattle feed and the milk disinfecting process [3]. Consequently, iodine deficiency has been reported in individuals adhering to strict vegan diets or diets excluding dairy products [4–6]. Additional cases have described iodine deficiency in the context of restrictive dietary patterns, including food allergies and avoidance of iodized salt [7, 8]. These reports suggest that iodine deficiency may occur even in regions traditionally considered iodine sufficient.

We present a case of severe hypothyroidism secondary to iodine deficiency in a toddler with multiple food allergies, including cow’s milk allergy. This case highlights the importance of careful dietary assessment and consideration of micronutrient deficiencies in pediatric patients with restrictive diets and growth abnormalities.

## 2. Case Presentation

A 23‐month‐old male was born full term to healthy parents, with no known family history of thyroid disease, food allergies, or immune deficiencies. The patient had previously been evaluated by a nutritionist at 9 months of age after developing multiple immunoglobulin E–mediated food allergies, including cow’s milk, eggs, and peanuts.

During infancy, the patient experienced multiple viral illnesses, including one episode of dehydration associated with gastroenteritis, had recurrent ear infections, and had ongoing difficulty with weight gain. At his initial nutrition telehealth visit, dietary history revealed significant dietary restrictions related to food allergies and caregivers expressed difficulty identifying appropriate meal options. The nutritional assessment concluded that the patient had increased energy needs related to recent illness and dietary limitations, as evidenced by poor weight gain. The recommended intervention included provision of three meals and two to three snacks per day, along with nutritional education for the parents.

At the patient’s 2‐year well‐child visit, the primary care provider noted only a minimal increase in height between 18 and 24 months of age (Figure 1) and ordered broad laboratory evaluation in addition to the routine two‐year screening. Thyroid function testing performed at this time revealed severe hypothyroidism, with markedly elevated TSH levels (204.81 mIU/L) and suppressed free thyroxine (free T4) levels (0.37 ng/dL) (Table 1).

**FIGURE 1:**
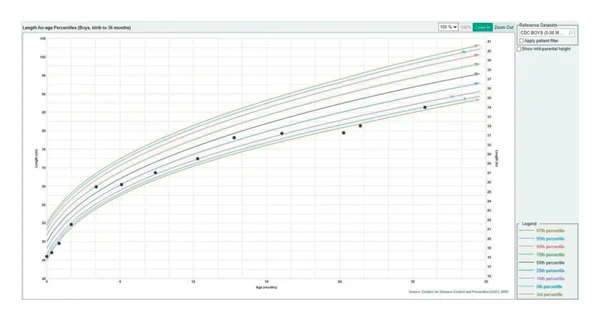
Length‐for‐age growth chart demonstrating plateau in linear growth between 18 and 24 months, preceding the diagnosis of hypothyroidism due to iodine deficiency.

**TABLE 1:** Diagnostic and follow‐up findings.

Parameters (reference range)	Baseline (prior to treatment)	Treatment start^∗^	One week posttreatment	One month posttreatment	Six months posttreatment
TSH (0.70–5.97 mIU/L)	204.81 ↑	—	18.2 ↑	4.57	4.41
Free T4 (0.85–1.75 ng/dL)	0.37 ↓	—	1.15	—	1.49
Thyroglobulin (1.7–55.6 ng/mL)	—	—	1591.2 ↑	—	—
Urinary iodine: creatinine (> 100 µg/g)	—	12.9 ↓	—	—	112.1
Thyroid antibodies (Negative)	Negative	Negative	Negative	—	Negative
25‐Hydroxyvitamin D (30–100 ng/mL)	—	—	23.5 ↓	—	37.5
Right thyroid lobe (age‐adjusted norms)	—	3.1 × 1.3 × 1.2 cm	—	—	2.7 × 1.1 × 0.9 cm
Left thyroid lobe (age‐adjusted norms)	—	3.2 × 1.2 × 1.7 cm	—	—	2.4 × 0.9 × 0.9 cm
Isthmus thickness (≤ 2 mm)	—	4.0 ↑	—	—	1.9

^∗^Treatment was levothyroxine at a dose of 25 mcg per day and a multivitamin containing 75 mcg of iodine. Levothyroxine and iodine treatment was initiated on the date of the thyroid ultrasonography appointment. Levothyroxine treatment discontinued 6 months later, and iodine treatment remains ongoing.

The patient was referred to pediatric endocrinology and evaluated one week after the 2‐year appointment. Physical examination revealed a diffusely enlarged thyroid gland, estimated to be greater than twice normal size and consistent with goiter; the gland was nontender on palpation. Additional laboratory testing demonstrated persistent elevation of TSH (18.2 mIU/mL), markedly elevated thyroglobulin (1591.2 ng/mL), and a low urinary iodine‐to‐creatinine ratio of 12.9, consistent with iodine deficiency (Table 1). Thyroid peroxidase and thyroglobulin antibodies were negative. The patient was also noted to have a low 25‐hydroxyvitamin D level (23.5 ng/mL) (Table 1).

Thyroid ultrasonography performed two days later demonstrated diffuse thyroid enlargement, with the right lobe measuring 3.1 × 1.3 × 1.2 cm and the left lobe measuring 3.2 × 1.2 × 1.7 cm. The isthmus was mildly thickened at 4 mm. No solid or cystic lesions were identified; however, pronounced hypervascularity was observed throughout the thyroid gland.

Treatment with levothyroxine at a dose of 25 mcg daily was initiated on the date of the thyroid ultrasonography appointment. Supplementation of 20 mcg/day vitamin D was also initiated, along with calcium (700 mg/day spread out over 2‐3 servings), and a multivitamin containing 75 mcg of iodine. Follow‐up laboratory testing one week later demonstrated normalization of free T4 (1.15 ng/dL) and TSH normalized to 4.57 mIU/mL after one month. Vitamin D levels normalized within days of supplementation. Subsequent endocrinology evaluation identified iodine deficiency as the underlying etiology of the patient’s hypothyroidism based on the low urinary iodine‐to‐creatinine ratio and elevated thyroglobulin levels. In addition to avoiding dairy and eggs, review of the patient’s diet suggested that commercially available allergy‐free foods consumed by the patient did not consistently contain iodized salt. This was further exacerbated by minimized iodized salt use in the home. The family was advised to incorporate iodized salt into the diet, and no other dietary modifications were implemented.

Six months posttreatment, laboratory studies demonstrated evidence of iodine repletion, with a urinary iodine‐to‐creatinine ratio of 112.1 (Table 1). Thyroid function tests remained within normal limits (free T4, 1.49 ng/dL; TSH, 4.41 mIU/mL). Repeat thyroid ultrasonography demonstrated interval reduction in the thyroid size, with the right lobe measuring 2.7 × 1.1 × 0.9 cm, the left lobe measuring 2.4 × 0.9 × 0.9 cm, and the isthmus measuring 1.9 mm in thickness (Table 1). Sonographic grayscale imaging was consistent with resolution of thyroid enlargement. Findings were considered markedly improved and consistent with iodine repletion and resolution of hypothyroidism. Levothyroxine was then discontinued.

At the patient’s 3‐year visit, after 1 year of iodine supplementation, the patient demonstrated sustained resolution of hypothyroidism and thyroid enlargement, with no ongoing clinical concerns. The patient continues to take the multivitamin containing 75 mcg of iodine.

## 3. Discussion

This case describes a toddler with severe hypothyroidism secondary to iodine deficiency as a consequence of a restrictive diet. Despite iodine deficiency being uncommon in high‐income countries with iodization programs, this case highlights the fact that pediatric iodine deficiency remains clinically relevant in children with restrictive diets, particularly dairy‐free diets, who do not consume iodized table salt. The patient’s presentation underscores the importance of dietary history and growth monitoring in the early recognition of endocrine disorders.

Iodine is essential for thyroid hormone synthesis, and deficiency results in the decreased production of thyroxine and triiodothyronine, leading to compensatory elevation of TSH and thyroid hypertrophy. In this patient, biochemical findings were notable for markedly elevated TSH levels exceeding 200 mIU/L, low FT4, and a substantially elevated thyroglobulin concentration, consistent with prolonged thyroid stimulation. Ultrasonography demonstrated diffuse thyroid enlargement with hypervascularity, further supporting the diagnosis of iodine deficiency–associated goiter. The absence of thyroid autoantibodies made autoimmune thyroid disease unlikely.

The diagnostic delay in this case reflects the nonspecific early manifestations of iodine deficiency in young children. Growth deceleration was the primary clinical sign prompting further evaluation, rather than classic symptoms of hypothyroidism [8]. Linear growth arrest between 18 and 24 months ultimately led to thyroid function testing and referral to endocrinology. This emphasizes the importance of routine growth surveillance and consideration of thyroid dysfunction in toddlers with unexplained growth failure, even in the absence of overt clinical symptoms.

Dietary factors played a central role in the development of iodine deficiency in this patient. The child’s multiple immunoglobulin E–mediated food allergies significantly restricted dietary options, and subsequent review revealed reliance on commercially available allergy‐free foods that did not consistently contain iodized salt. While iodine sufficiency is often assumed in regions with salt iodization programs (in the United States, this program is voluntary), recent reports suggest that certain specialty diets, including allergy‐restricted, plant‐based, and processed food–based diets, may be insufficient in iodine [9, 10]. This case reinforces the need for clinicians to assess iodine intake in children with dietary restrictions and to remain cautious about assuming iodine adequacy.

Treatment with levothyroxine resulted in rapid normalization of thyroid hormone levels, and subsequent laboratory and imaging follow‐up demonstrated the resolution of biochemical hypothyroidism, goiter, and iodine deficiency. Improvement in urinary iodine‐to‐creatinine ratio and marked reduction in thyroid gland size on follow‐up ultrasonography were consistent with iodine repletion. These findings suggest that iodine deficiency–associated hypothyroidism in early childhood may be reversible when promptly identified and treated although prolonged deficiency carries risks for neurodevelopmental impairment.

While prior cases of iodine deficiency–induced hypothyroidism have been reported, this case remains unique in the circumstances that led to the deficiency, specifically the combination of the age of onset and corresponding dietary restriction. Two cases in preadolescents have demonstrated iodine deficiency–induced hypothyroidism secondary to restrictive diets, either related to autism‐associated food selectivity or dietary management of eosinophilic esophagitis [11, 12]. Other reports have highlighted hypothyroidism due to strict vegan or vegetarian diets [4–6]. Leniszewski et al. described a 12‐year‐old boy with a seafood allergy who developed iodine deficiency after eliminating all seafood at age 6 and switching to noniodized sea salt at age 10 [7]. Table 2 highlights the key comparisons between current literature on cases of iodine deficiency–induced hypothyroidism.

**TABLE 2:** Reported cases of dietary restriction causing iodine‐deficiency hypothyroidism.

Study	Age	Dietary restriction	Key feature	Outcome
Kanaka et al. [4]	7.5 months	Vegan diet	Goiter, hypothyroidism	Improved
Patel et al. [13]	5 years	Texture sensitivities	Goiter, hypothyroidism	Improved
Yeliosof et al. [5]	2 years	Dairy‐free diet	Hypothyroidism	Improved
Sasidharan and Quintos [14]	6 years	Vegan diet	Goiter, hypothyroidism	Resolved
Brooks and Post [10]	9 years	Avoidance of eggs, grains, and milk to manage eosinophilic esophagitis	Goiter, hypothyroidism	Resolved
Pillai and Quintos [6]	12 years	Seafood allergy, use of non‐iodized salt alternative	Severe hypothyroidism	Improved
Patel et al. [13]	12 years	AIP (Autoimmune Protocol) diet, (no legumes, no gluten, no dairy, no seeds, no grains, and no nightshades)	Moderate hypothyroidism	Improved
Patel et al. [13]	12 years	Kosher and Himalayan salt use with iodized salt restricted to baking products.	Severe hypothyroidism, thyromegaly	Improved
Croce et al. [9]	13 years	Autism, restrictive diet	Goiter, hypothyroidism	Improved
Present case	23 months	Cow’s milk allergy; noniodized allergy‐free foods	Growth arrest, goiter	Resolved

In contrast, the present case involves a younger child whose iodine deficiency resulted from a cow’s milk allergy and an egg allergy. The child’s parents also minimized the use of iodized table salt. This case illustrates that restrictive diets due to food allergies can lead to early‐onset iodine deficiency, highlighting a population that may be under‐recognized and at risk for severe hypothyroidism and growth delay.

## 4. Conclusion

This case demonstrates that iodine deficiency should remain in the differential diagnosis of severe hypothyroidism and growth delay in young children, even in regions where iodine sufficiency is generally presumed. Restrictive diets due to food allergies may place toddlers at a particular risk, especially when iodized salt and iodine‐rich foods are limited. Careful dietary assessment, routine growth monitoring, and targeted laboratory evaluation are essential for early recognition and treatment. Increased awareness of iodine deficiency in pediatric patients with restrictive diets, including those with multiple food allergies, may help prevent delayed diagnosis and associated developmental consequences.

## Funding

No funding was received for this manuscript.

## Consent

Written informed consent was obtained from the legal guardians of the patient presented in this publication.

## Conflicts of Interest

The authors declare no conflicts of interest.

## Data Availability

The data that support the findings of this study are available from the corresponding author upon reasonable request.
